# Surface-enhanced Raman spectroscopy of cell lysates mixed with silver nanoparticles for tumor classification

**DOI:** 10.3762/bjnano.8.120

**Published:** 2017-06-01

**Authors:** Mohamed Hassoun, Iwan W.Schie, Tatiana Tolstik, Sarmiza E Stanca, Christoph Krafft, Juergen Popp

**Affiliations:** 1 Leibniz Institute of Photonic Technology, Albert Einstein Str. 9, 07745 Jena, Germany; 2 Institute of Physical Chemistry & Abbe Center of Photonics, Friedrich Schiller University Jena, Helmholtzweg 4, 07743 Jena, Germany; 3 Department of Internal Medicine IV, Division of Gastroenterology, Hepatology and Infectious Diseases, Jena University Hospital, Erlanger Allee 101, 07745 Jena, Germany

**Keywords:** cell lysate, silver nanoparticles, surface-enhanced Raman spectroscopy (SERS), tumor-cell differentiation

## Abstract

The throughput of spontaneous Raman spectroscopy for cell identification applications is limited to the range of one cell per second because of the relatively low sensitivity. Surface-enhanced Raman scattering (SERS) is a widespread way to amplify the intensity of Raman signals by several orders of magnitude and, consequently, to improve the sensitivity and throughput. SERS protocols using immuno-functionalized nanoparticles turned out to be challenging for cell identification because they require complex preparation procedures. Here, a new SERS strategy is presented for cell classification using non-functionalized silver nanoparticles and potassium chloride to induce aggregation. To demonstrate the principle, cell lysates were prepared by ultrasonication that disrupts the cell membrane and enables interaction of released cellular biomolecules to nanoparticles. This approach was applied to distinguish four cell lines – Capan-1, HepG2, Sk-Hep1 and MCF-7 – using SERS at 785 nm excitation. Six independent batches were prepared per cell line to check the reproducibility. Principal component analysis was applied for data reduction and assessment of spectral variations that were assigned to proteins, nucleotides and carbohydrates. Four principal components were selected as input for classification models based on support vector machines. Leave-three-batches-out cross validation recognized four cell lines with sensitivities, specificities and accuracies above 96%. We conclude that this reproducible and specific SERS approach offers prospects for cell identification using easily preparable silver nanoparticles.

## Introduction

Cytopathology is the histopathologic inspection of cells. Dyes, such as hematoxylin for cell nuclei or eosin for cytoplasm, are commonly used to stain cells with subsequent microscopic assessment by pathologists. Complementary tools are immunocytochemistry, which uses fluorescence-labeled antibodies against cellular antigens, and flow cytometry, which combines several detection channels based on light scattering, absorption and fluorescence with microfluidic flow systems.

Raman spectroscopy has been proposed as promising technique for cell characterization and cell identification because of its high chemical specificity under label-free and non-destructive conditions [1–2]. Raman spectroscopy is based on inelastic light scattering from molecular bonds. It probes the molecular vibrations of all cellular biomolecules, such as nucleic acids, proteins, lipids and carbohydrates and provides chemical fingerprint spectra of cells. The throughput of spontaneous Raman spectroscopy for cell classification is limited to the range of one cell per second by the inherently low efficiency of the inelastic scattering process of photons and the resultant low signal intensity. Compared to modern flow cytometers with a throughput of thousands cells per second, this severely restricts the applicability of Raman spectroscopy in this field. This limitation can be overcome by signal-enhancement approaches including surface-enhanced Raman scattering (SERS), resonance Raman scattering, coherent anti-Stokes Raman scattering and stimulated Raman scattering [3]. For the analysis of liquids, SERS is the most frequently applied approach and has been used for analyte detection in the submicromolar range [4–5]. SERS fingerprint spectra of molecules are generated when incident light excites localized surface plasmons on nanometer-sized metallic structures. A strong electromagnetic field is then created near the metallic surface and enhances the Raman scattering of nearby molecules. The plasmonic properties of SERS-active nanoparticles depend on the preparation conditions, the type of metal, the size and the shape of these nanoparticles [6–10], and their aggregation state [11–12]. Increasing the size of nanoparticle aggregates shifts the excitation wavelength to the near-IR region and therefore longer excitation wavelengths can be used for SERS measurements.

SERS was also suggested for cell identification [13–14]. While the signal intensity is similar to that of fluorescence emission, SERS nanoparticles do not suffer from photobleaching and offer a high multiplex capability due to narrow band widths. Enhancement of Raman signal of cells can be realized by (1) various techniques of nanoparticles delivery into cells, such as spontaneous uptake, microinjection, electroporation [15–20] or (2) binding of antibody-functionalized nanoparticles to specific antigens [21–23]. The disadvantages of approach (1) include the poor reproducibility due to nonspecific binding of nanoparticles, the long time needed for nanoparticles uptake by cells, and the heterogeneity of nanoparticles inside cells. Approach (2) is complicated because of complex protocols for nanoparticle preparation with Raman reporters, protective shells and antibodies. Furthermore, approach (2) cannot be considered to be label-free anymore. In the context of microbial identification, bacterial cells were lysed by sonication, and the bacterial lysate were mixed with nanoparticles to allow interaction between nanoparticles and bacterial biomolecules [24]. This gave very reproducible SERS spectra.

The current study transfers this SERS approach to distinguish four human cancer cell lines. These cell lines are two liver cancer cell lines (HepG2 isolated from liver tissue of a male patient with well differentiated hepatocellular carcinoma and SK-Hep1 received from ascetic fluid of a patient with adenocarcinoma of the liver), one breast cancer cell line (MCF-7 obtained from a female patient) and one human pancreatic ductal adenocarcinoma cell line (Capan-1). A protocol was developed to disrupt the cell walls by sonication and to allow for the interaction of silver nanoparticles with the released cellular biomolecules. The measured SERS spectra from six different batches were subjected to a support vector machine (SVM) to train classification models. The sensitivities, specificities and accuracies of the SVM model were calculated by cross-validation schemes. This proof-of-principle demonstrates that non-functionalized, easy-to-prepare silver nanoparticles give reproducible SERS spectra that can be used for the identification of human cancer cells.

## Results and Discussion

**Figure 1:**
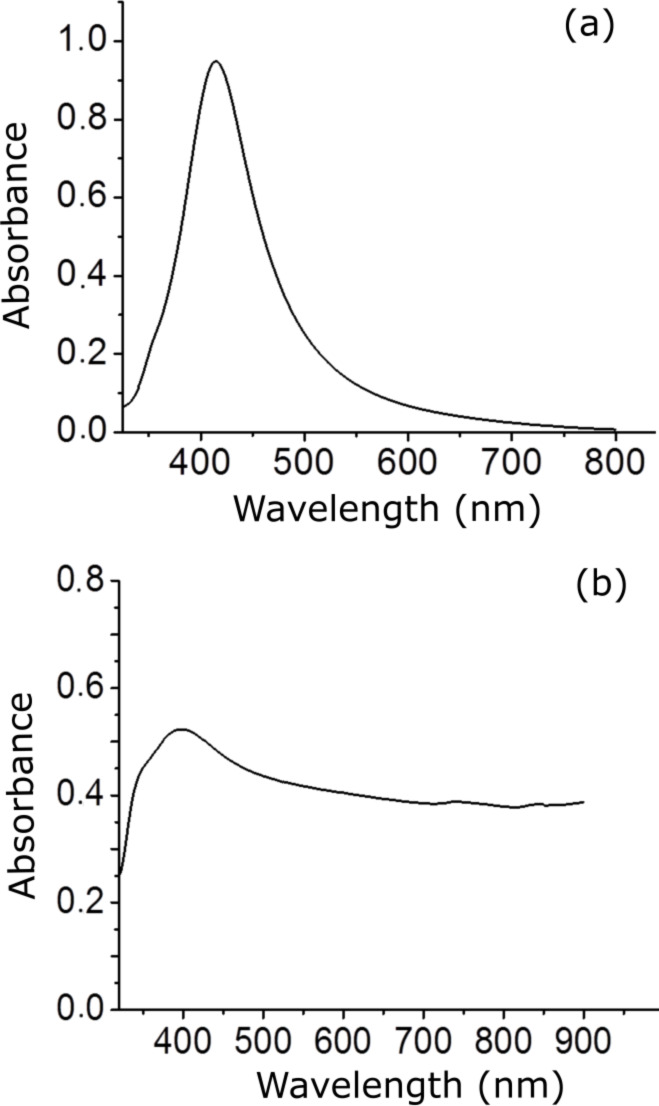
UV–vis absorption spectra of (a) silver nanoparticles with an absorption band at 415 nm and (b) solution of silver nanoparticles and potassium chloride. The absorption band of aggregated nanoparticles was shifted to near infrared region.

The absorption band of silver (Ag) nanoparticles corresponds to the maximum of the plasmon resonance which is near 415 nm (Figure 1a). Shifting the plasmon resonance of our nanoparticles to the near-IR spectral region was achieved by aggregation using potassium chloride (KCl). When nanoparticles aggregate, they become electronically coupled, which results in a change of the surface plasmon resonance compared to individual particles. Figure 1b shows the effect of adding KCl to Ag nanoparticles on the optical absorption characteristics. The aggregated nanoparticles have a broad absorption band that allowed for SERS measurements with an excitation laser at 785 nm.

**Figure 2:**
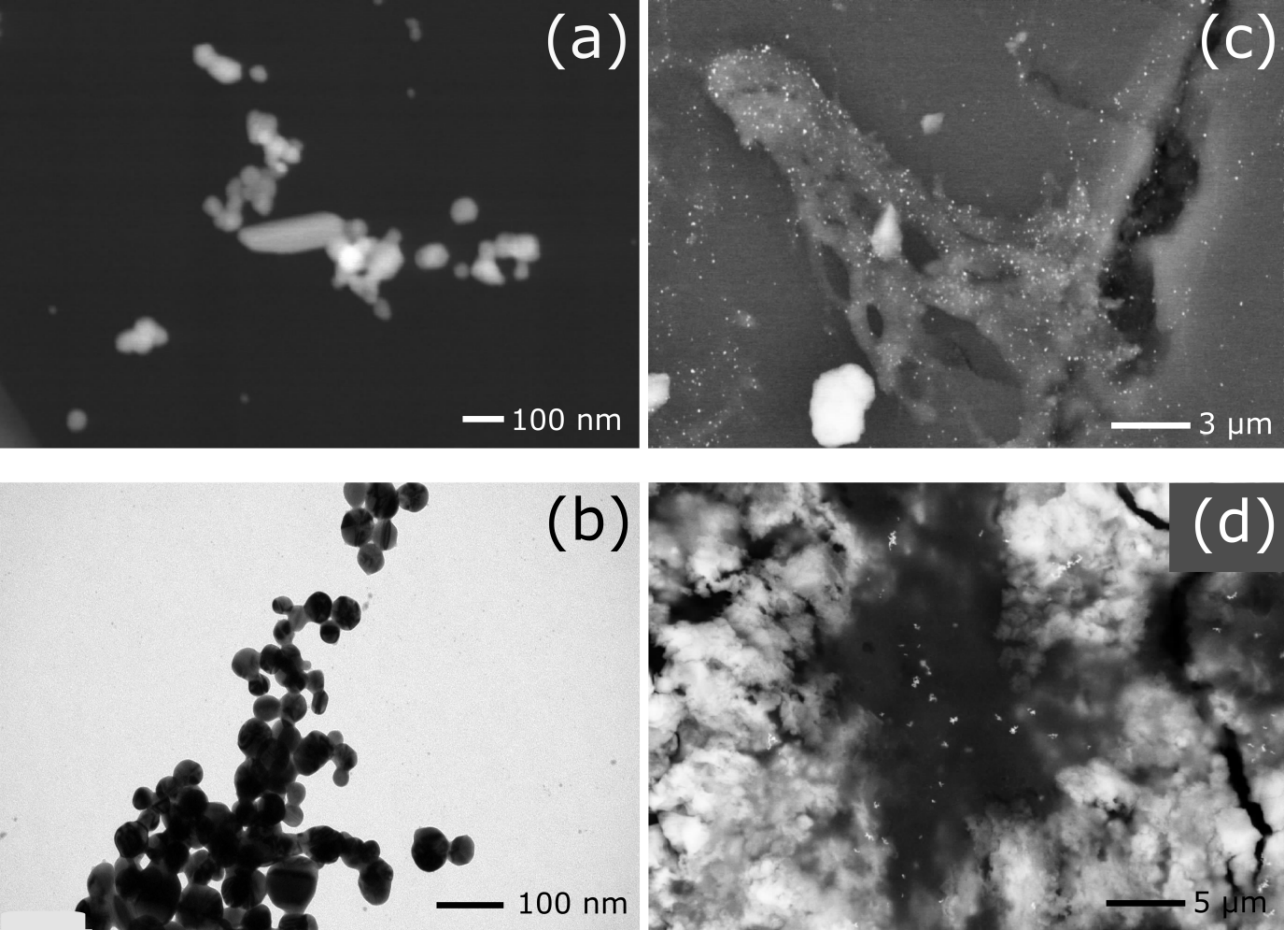
(a) Scanning electron microscopy (SEM) image of silver nanoparticles. The nanoparticles have a high degree of polydispersity in size ranging from 10 to 100 nm with an average size close to 50 nm. (b) Transmission electron microscopy image of silver nanoparticles showing their predominantly spherical shape and polydispersity in size. (c) SEM image of intact cells mixed with nanoparticles showing the distribution of nanoparticles on the surface of the cell. (d) SEM image of cell lysate mixed with nanoparticles showing released cellular biomolecules with nanoparticles after disruption of cell membrane.

The size and shape of Ag nanoparticles were also analyzed by electron microscopy. Transmission electron microscopy (TEM) images and scanning electron microscopy (SEM) images of silver nanoparticles are compared in Figure 2a and b. The average size of the Ag nanoparticles was determined to be around 50 nm with a high degree of polydispersity in size ranging from 10 to 100 nm. The Ag nanoparticles do not tend to aggregate to a single specific shape after adding KCl. Instead, they form different shapes from spheres to rods. The cells, before and after sonication, were mixed with Ag nanoparticles and SEM images were recorded to better understand the diffusion of nanoparticles inside the cells. Nanoparticles represented by light spots are shown on the surface of a cell wall in Figure 2c and during interaction with cellular biomolecules in Figure 2d.

**Figure 3:**
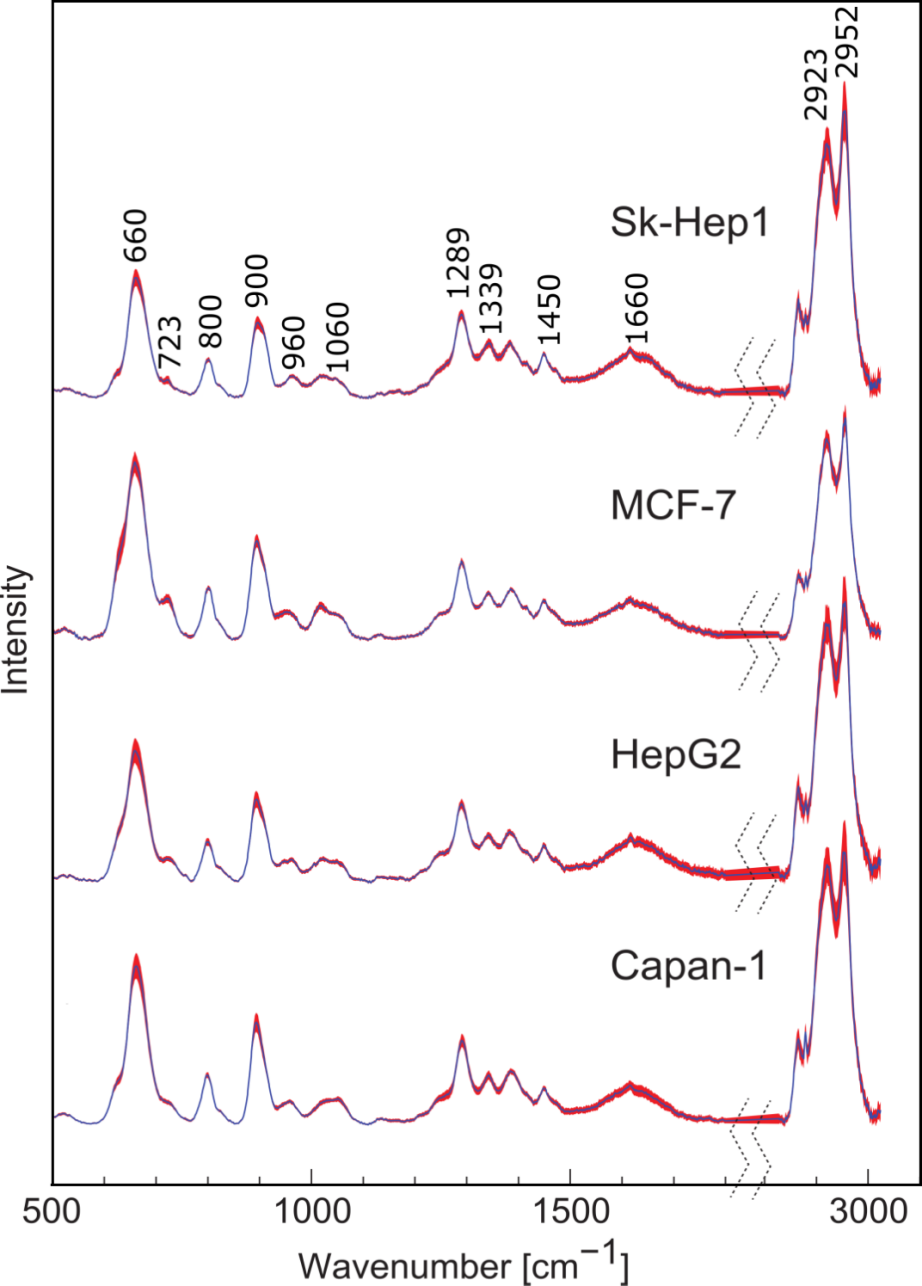
Preprocessed mean SERS spectra and standard deviations of the different cell lines. Labeled bands are assigned to cellular biomolecules including nucleic acids, proteins and carbohydrates. The low standard deviation values (represented by the red shadow) emphasize the high reproducibility of the technique.

Cellular biomolecules including nucleic acids, proteins, carbohydrates and lipids are released after disruption of the cell membranes and can interact with nanoparticles. The spectral bands obtained from SERS measurements can then be assigned to biomolecules of cell nucleus and the cytoplasm. The raw spectra were baseline-subtracted and normalized. Figure 3 shows the processed mean SERS spectra and the standard deviation for each of the four cell lines Capan-1, HepG2, MCF-7 and Sk-Hep1. The band at 660 cm^−1^ is assigned to carboxylate [25]. Spectral contributions of adenine from nucleic acids and metabolites appear at 723 and 1339 cm^−1^ and can be assigned to adenine ring-breathing modes [18,26–27]. Protein vibrations contribute to the band at 900 cm^−1^. The bands at 800 and 960 cm^−1^ can be assigned to CN stretching vibrations. Carbohydrates are represented by bands in the spectral region of 1000–1100 cm^−1^. The bands at 1289 cm^−1^ and 1660 cm^−1^ can be assigned to the amide III and amide I vibrational modes of peptide bonds in proteins, respectively [18,26,28]. The band at 1450 cm^−1^ arises from CH_2_ deformation vibrations of all biomolecules. The bands at 2923 and 2952 cm^−1^ can be assigned to CH_2_ and CH_3_ stretching vibrations of all biomolecules [24,26,28]. The reproducibility of these spectra was tested by measuring the SERS spectra from six batches of the four cell lines. The small standard deviation values proved the high reproducibility.

**Figure 4:**
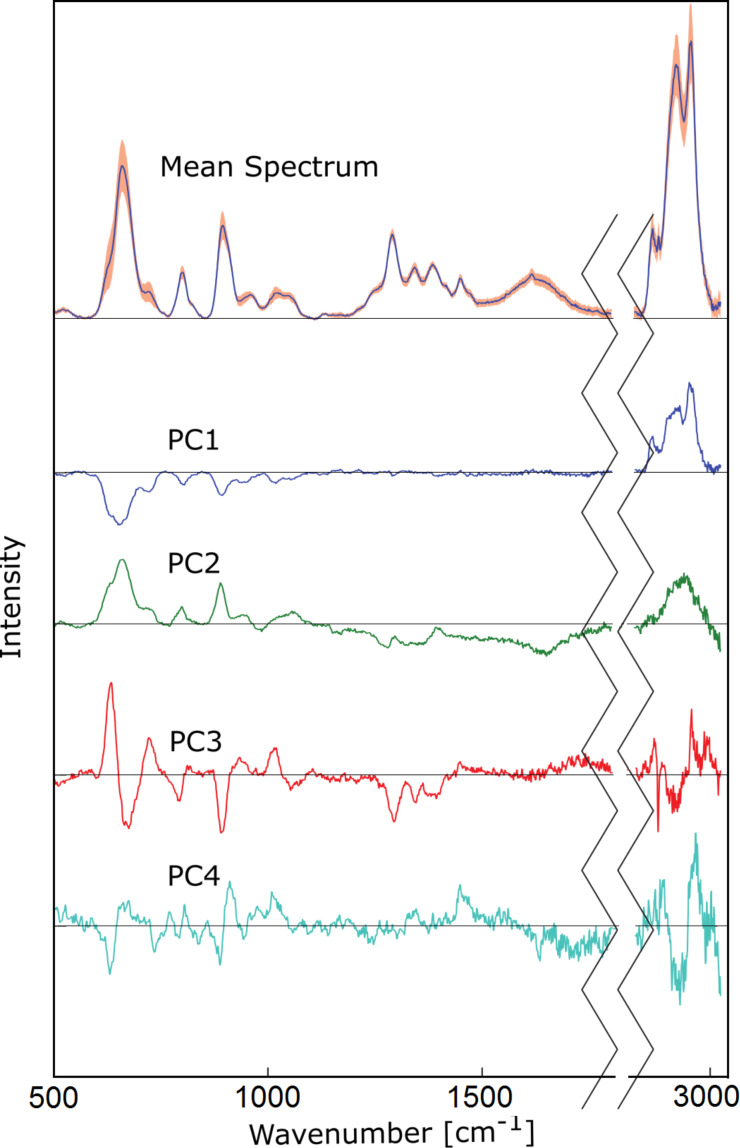
First four principal components used for the support vector machine model. These loadings represent 89% of data variance between MCF-7, Capan-1, SK-Hep1 and HepG2 cell lines.

It is evident from Figure 3 that the SERS spectra of the individual cell lines are highly similar and the cell lines cannot easily be distinguished by univariate analysis of single bands or band ratios. Therefore, multivariate classification was applied for differentiation of the cell lines. Prior to multivariate classification the data size was reduced by principal component analysis (PCA). Figure 4 shows the first four principal components (PCs) that described 89% of the variances of the data set required for cell line differentiation. PC1 loadings showed negative bands in the fingerprint range from 600 to 1200 cm^−1^ and positive signals from 2800 to 3000 cm^−1^. The most pronounced spectral features were (i) positive bands near 660, 900 and 2900 cm^−1^ in PC2 loadings, (ii) a derivative-like feature at 660 cm^−1^ and negative bands near 723 and 1339 cm^−1^ in PC3 loadings, and (iii) negative band near 660 and derivative-like feature near 900 cm^−1^ in PC4 loadings. In general, we did not notice a significant difference in the amide content inside the four cell lines. The main differences were assigned to vibrations of nucleic acids, CH_2/3_ from the whole cell contents and the carboxylate moieties.

**Figure 5:**
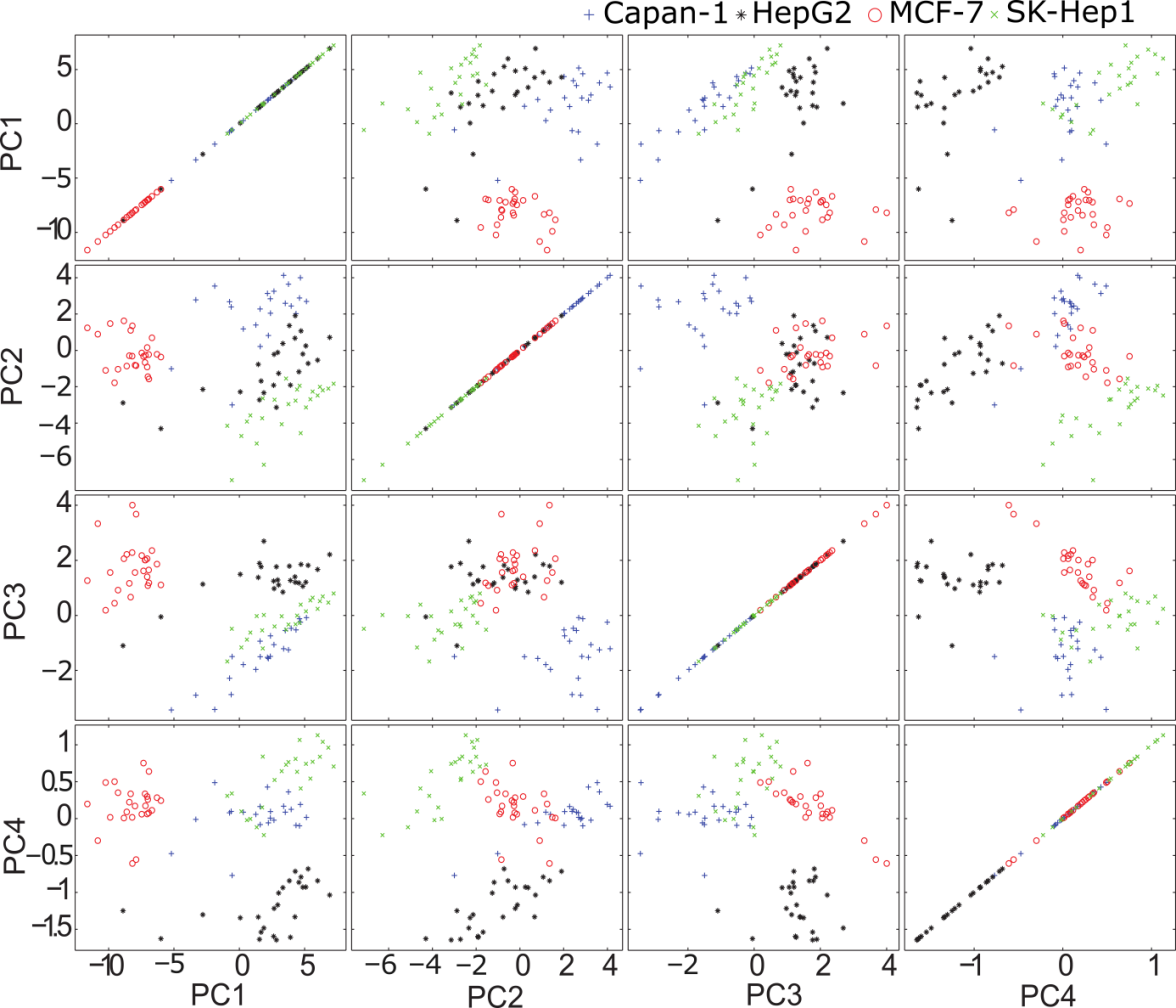
Score values of first four principal components of different cell lines. The four cell lines, MCF-7 (red circle), Capan-1 (blue plus sign), SK-Hep1 (green cross) and HepG2 (black star) are distinguished based on the first four PCs.

The score values of the first four PCs are plotted in Figure 5. Based on four PCs the main variations between the four cell lines were explained, and cells could be differentiated. Negative PC1 scores separated the spectra of the MCF-7 cell line from the spectra of the other cell lines having positive PC1 score values. PC2, PC3 and PC4 distinguished Capan-1, SK-Hep1 and Hep-G2.

The first four PCs were used as input for classification based on support vector machines (SVM). The SVM model was trained with three batches of cell lines and then tested with three different batches of the same cell lines. This allowed for 20 different batch permutations for validation and gave a reliable unbiased classification model. The test was run 20 times and the sensitivity, specificity and accuracy of the SVM model in each run were calculated. Table 1 shows the number of spectra that were classified correctly for each cell line in the 20 tests. Of 939 trial tests of Capan-1 spectra, the SVM model was able to identify the spectra correctly as Capan-1 cells 906 times with 96.7% accuracy. In case of Hep-G2 cells the model was able to correctly identify the spectra 898 times out of 1005 trials with 97.1% accuracy. The MCF-7 cell line was identified correctly in all 932 test trials with a very high accuracy of 99.1%. The identification of Sk-Hep1 cell line was true in 980 times out of 1002 trials with 98.8% accuracy.

**Table 1:** Results of the identification of different cell lines. The support vector machine model (SVM) model was trained with spectra taken from three different batches of each cell line and tested with data taken from the remaining three batches. The SVM model was run for 20 different permutations.

sample cell line	identified by SVM as			
	Capan-1	HepG2	MCF-7	SK-Hep1

Capan-1	906	6	3	24
HepG2	74	898	33	0
MCF-7	0	0	932	0
SK-Hep1	22	0	0	980

Table 2 summarizes the mean values of the sensitivity, specificity and accuracy plus the deviation of each value. The highest mean sensitivity value of 100% was obtained in the case of MCF-7 cell line as the PC1 includes most of information about variations between MCF-7 cells versus Capan-1, HepG2 and SK-Hep1 cells. The lowest sensitivity value was obtained in the case of HepG2 cell line with 89.4%. The maximum and minimum mean specificity values are 99.8% in case of HepG2 and 96.7% in case of Capan-1. These results confirm the ability to detect the molecular variations between the different tumor cell lines based on the SERS spectra of cell lysates mixed with nanoparticles and SVM-based classification.

**Table 2:** Mean sensitivity, specificity and accuracy values of support vector machine model for each cell line (in percentage).

	cell line			
	Capan-1	HepG2	MCF-7	SK-Hep1

mean sensitivity %	96.5 ± 4.4	89.4 ± 10.5	100	97.8 ± 2.9
mean specificity %	96.7 ± 3.7	99.8 ± 0.5	98.8 ± 1.7	99.2 ± 1.2
accuracy %	96.7	97.1	99.1	98.8

## Conclusion

Four different human tumor cell lines, Capan-1, HepG2, Sk-Hep1 and MCF-7, were lysed using ultrasonication and then mixed with aggregated silver nanoparticles. The reproducibility of SERS spectra was demonstrated by preparing six batches and measuring them under the same conditions. The values of standard deviation, calculated for different batches, were small. PCA was performed to reduce the size of the data and assess variations between the four cell lines. Four PCs were used as input to a SVM model to classify these cell lines. Leave-three-batches-out cross validation was performed to test the stability of the SVM model. The SVM model was able to identify the different cell lines from each other with very high accuracy, sensitivity and specificity. The accuracy values were 96.7%, 97.1%, 99.1% and 98.8% for identification of Capan-1, HepG2, MCF-7 and Sk-Hep1, respectively. These values agree with classification results based on Raman spectra [29]. Compared to Raman spectra of intact cells, the SERS spectra of cell lysates contain fewer bands whose intensities are enhanced. More importantly, the variations in SERS spectra between different cells are also enhanced that contribute to accurate and stable classification.

The presented approach is a rapid, easy, efficient, highly reliable and specific strategy to identify and classify different human cancer cell lines without need for complex sample preparation procedures. To reduce the sample volume and measurement time towards few milliseconds, and automate mixing of solvents and acquisition of SERS spectra, this approach will be transferred to a droplet-based microfluidic lab-on-chip device [24]. After delivery of non-functionalized nanoparticles into cells [20], the SERS approach can also increase the throughput of tumor cell recognition in microfluidic chips at continuous flow [30]. With exposure times in the millisecond range, SERS assessment of millions of cells comes within reach in the future. A possible scenario for screening of millions of blood cells and enumeration of rare circulating tumor cells in blood of cancer patients is a combination of all approaches mentioned above: generation of droplets with single cells in a microfluidic chip, addition of cell lysis buffer, nanoparticles and activation salt, mixing of all solvents and collection of SERS spectra for classification.

## Experimental

### Nanoparticle preparation

Silver nitrate (ACS reagent, ≥99%), sodium hydroxide, hydroxylamine hydrochloride (reagent plus, 99%) and potassium chloride were purchased from Sigma–Aldrich. Distilled water was used for all preparations. The silver nanoparticle colloids were synthesized according to the protocol described by Leopold and Lendl [31]. Briefly, 1 mM silver nitrate was added to a solution of 1.5 mM hydroxylamine hydrochloride and 3 mM sodium hydroxide. The whole mixture was stirred during the addition of the silver nitrate. As a sign of a successful preparation the color of the solution changed from grey to yellow. The silver colloids were then preserved in the refrigerator at 4 °C. 1 M of KCl was prepared in distilled water. The preparation procedure can be performed quickly and at room temperature.

### Nanoparticle characterization

**Transmission electron microscopy (TEM):** 5 µL of the particle dispersion were deposited on a carbon-coated 400 mesh copper grid. After 1 min of adsorption the excess liquid was blotted off with filter paper. Dried samples were then examined by a JEM 1400 (JEOL, Tokyo, Japan) transmission electron microscope.

**Scanning electron microscopy (SEM):** Measurements were performed by a field emission microscope JSM-6300F (JEOL, Tokyo, Japan). The energy of the exciting electrons was 5 keV. Beside the detector for secondary electrons (SEI) the system is equipped with different detector types (semiconductor and YAG) for backscattered electrons.

**Spectrophotometry:** The UV–vis spectra of silver nanoparticles and KCl-aggregated silver nanoparticles were measured in the spectral range of 200–800 nm with a Jasco V-670 diode UV–vis spectrophotometer (Hachioji, Tokyo, Japan) using plastic cuvettes (Brand GmbH Wertheim Germany) of 1 cm light path.

### Cell cultivation

Liver cancer cell lines (HepG2 and SK-Hep1) were cultivated in RPMI 1640 liquid medium with 20 mM HEPES, stable glutamine (FG 1235, Biochrom AG, Germany), 10% fetal bovine serum (10099-133, Life Technologies, Germany) together with 100 units/mL of penicillin and 100 µg/mL of streptomycin (15140, Gibco^®^, Life Technologies GmbH, Germany). Cultivation of MCF-7 breast cancer cells was performed in RPMI 1640 with 2.0 g/L NaHCO_3_ (F 1215, Biochrom AG, Germany) and 40 mg/L folic acid (F7876, Sigma–Aldrich, Germany) with the same amount of fetal bovine serum, penicillin and streptomycin as described above for liver cells. The pancreatic cancer cell line Capan-1 was cultured in IMDM medium (12440-053, Life Technologies, Germany) complemented with 20% fetal bovine serum (10099-133, Life Technologies, Germany), 100 units/mL of penicillin and 100 µg/mL of streptomycin (15140, Gibco^®^, Life Technologies GmbH, Germany). The cells were maintained in an incubator at 37 °C, 90% humidity and 5% carbon dioxide in air. 75 cm^2^ cell culture flasks (658170; Greiner Bio-One GmbH, Germany) were used for cultivation of the cell lines. Every two or three days the medium was changed until approximately 100% confluence was reached. Cells were detached from the substrate by a 0.05% of trypsin–EDTA solution (L2143; Biochrom AG, Germany) and fast frozen at −20 °C. The final number of cells in each flask was around 10^7^ cells/mL, which was confirmed by cell counting, using Neubauer Chamber (0.0025 mm^2^; Marienfied, Germany). In order to prove the reproducibility of our experiments six batches of each of the four cell lines were prepared. The optical density of different cell lines were measured using Eppendorf Biophotometer plus. The optical density of 0.25 was correlated to an averaged cell number of 10^7^ cells/mL.

### Cell sonication

Cells were sonicated using an ultrasonic probe system (Brandelin SONOPULS HD 2070) with a maximum output power of 70 W. This sonication technique helps disrupting the cell membranes and allows for an interaction of released cell components with the silver nanoparticles. The probe was inserted inside an Eppendorf tube containing 1 mL of the cells in PBS solution. The sonication was applied in 3 cycles of 15 s each and 5 s break in between with a power set to 20%. The cell lysate was then transferred to a new tube and stored until further processing in a freezer.

### Raman spectroscopy and SERS measurements

SERS measurements were performed on a commercial Raman microscopy system (Holoprobe, Kaiser Optical system, USA). This system consists of a multi-mode diode laser with 785 nm excitation wavelength (Invictus NIR laser), an *f*/1.8 spectrograph with a holographic transmission grating (Kaiser Optical system, USA), and a Peltier-cooled back-illuminated deep-depletion CCD detector (iDus420, Andor, Ireland). The microscope was coupled to the Raman system with fibers of 65 µm core diameter. A 10×/0.25 objective lens (Leica, Germany) was used for all SERS measurements. The laser wavelength was calibrated using cyclohexane. The system was intensity calibrated using a white light source. The laser power was fixed at 50 mW with an acquisition time of 5 s. Each batch was lysed and divided into eight to ten samples. 100 µL of the silver nanoparticles were mixed with 100 µL KCl as aggregating agent, and then 100 µL of cell lysate were added to the mixture with a final ratio of 1:1:1. 200 µL solution was filled in vials that were cut from 0.2 mL 96-well thin wall thermal cycler plates, and the laser beam was focused on the surface of the mixture. One spectrum was collected from each sample. The experiments were repeated using six batches for each cell line and the reproducibility was tested by calculating the standard deviation from the mean spectra.

### Data analysis

The intensity-corrected SERS spectra were exported to Matlab (The Mathworks, USA) and pre-processed before the evaluation of the spectral classification models. The imported spectra were corrected for the dark current and the constant voltage bias by subtracting a smoothed dark spectrum. The resulting spectra were corrected for the polynomial background arising from residual excitation light using the penalized least squares-based Whittaker smoother algorithm outlined by Eilers [32]. The background corrected data was cropped to a low-wavenumber region between 500 and 1800 cm^−1^ and a high-wavenumber region between 2828 and 3028 cm^−1^. Both regions were combined and area-normalized relative to the spectral wavenumber region. Spectral classification was performed by support vector machines (SVM) with a linear kernel, using the libSVM Matlab library by Chang [33]. The classification was performed batch-wise; three batches were used to build a model and the remaining three batches were used for testing. With six total batches 20 different batch permutations were used for model building and for model testing. Before performing the SVM-based classification the dimensionality of the data set was reduced by principal component analysis (PCA) for the three batches, where on average the first four principal components (PCs) describe 89% of the data variance. The classification was performed on the score values of the first four PCs. After training the SVM model with the score values of the training batches the spectra of the test batches were projected onto the four loading vectors created by the training batches, and the resulting score values were used as the test set. The confusion matrices established after testing each batch permutation were summed up.
